# Retraction: Recent application of visible-light induced radicals in C–S bond formation

**DOI:** 10.1039/d4ra90096k

**Published:** 2024-09-05

**Authors:** Vishal Srivastava, Pravin K. Singh, Arjita Srivastava, Praveen P. Singh

**Affiliations:** a Department of Chemistry, CMP Degree College, University of Allahabad Prayagraj 211002 India; b Department of Chemistry, United College of Engineering & Research Naini Prayagraj 211010 India ppsingh23@gmail.com

## Abstract

Retraction of ‘Recent application of visible-light induced radicals in C–S bond formation’ by Vishal Srivastava *et al.*, *RSC Adv*., 2020, **10**, 20046–20056, https://doi.org/10.1039/D0RA03086D.

The Royal Society of Chemistry, with the agreement of the signed authors, hereby wholly retracts this *RSC Advances* review article due to significant portions of text overlap with a number of sources throughout the review article, in particular ref. 57, 60, 65, 68, 69, 70, 72, 73, 75, 78, 79, 80, 82 and 83 of the original article and ref. 1 below. Although many of these articles have been cited, it was not made clear that some of the text was reproduced from these articles.

Signed: Vishal Srivastava, Pravin K. Singh and Praveen P. Singh

Date: 29th August 2024

Retraction endorsed by Laura Fisher, Executive Editor, *RSC Advances*
